# Environmental phenol mixture during pregnancy and child sleep quality in the ECHO cohort

**DOI:** 10.3389/fped.2025.1533015

**Published:** 2025-08-08

**Authors:** Sarah D. Geiger, Xiaoshuang Xun, Cai Zhang, Aruna Chandran, Kritika Madan, Grace Kim, Fatima Naveed, Megan Woodbury, Dana E. Goin, Stephanie M. Eick, Courtney K. Blackwell, Maxwell Mansolf, Max Aung, Akram Alshawabkeh, Dana Dabelea, Anne L. Dunlop, Assiamira Ferrara, Jonika B. Hash, Monique Hedderson, Erica Jansen, Monique LeBourgeois, Louise O’Brien, Yeyi Zhu, Susan L. Schantz

**Affiliations:** ^1^ Beckman Institute for Advanced Science and Technology, University of Illinois at Urbana-Champaign, Urbana, IL, United States; ^2^ Department of Kinesiology and Community Health, University of Illinois at Urbana-Champaign, Urbana, IL, United States; ^3^ Department of Epidemiology, Johns Hopkins Bloomberg School of Public Health, Johns Hopkins University, Baltimore, MD, United States; ^4^ College of Engineering, Northeastern University, Boston, MA, United States; ^5^ Columbia Department of Epidemiology, Mailman School of Public Health, Columbia University, New York, NY, United States; ^6^ Gangarosa Department of Environmental Health, Emory University Rollins School of Public Health, Atlanta, GA, United States; ^7^ Department of Medical Social Sciences, Feinberg School of Medicine, Northwestern University, Chicago, IL, United States; ^8^ Keck School of Medicine, University of Southern California, Los Angeles, CA, United States; ^9^ Lifecourse Epidemiology of Adiposity and Diabetes (LEAD) Center, University of Colorado Anschutz Medical Campus, Aurora, CO, United States; ^10^ Department of Gynecology & Obstetrics, Emory University School of Medicine, Atlanta, GA, United States; ^11^ Center for Upstream Prevention of Adiposity and Diabetes Mellitus (UPSTREAM), Kaiser Permanente Northern California, Oakland, CA, United States; ^12^ Department of Child, Family, and Population Health Nursing, School of Nursing, University of Washington, Seattle, WA, United States; ^13^ Division of Research, Kaiser Permanente Northern California, Oakland, CA, United States; ^14^ Department of Nutritional Sciences, University of Michigan School of Public Health, Ann Arbor, MI, United States; ^15^ Department of Integrative Physiology, University of Colorado Boulder, Boulder, CO, United States; ^16^ Division of Sleep Medicine, Department of Neurology, Department of Obstetrics & Gynecology, University of Michigan, Ann Arbor, MI, United States

**Keywords:** child, environmental pollutants, phenols, sleep quality, pregnancy

## Abstract

**Introduction:**

Poor sleep quality in childhood can predict sleep quality throughout the lifecourse and other health outcomes. Endocrine-disrupting chemicals can affect adults’ sleep quality, and prenatal phenol exposure impacts fetal development.

**Objective:**

To assess associations between prenatal phenol concentrations and child sleep outcomes.

**Methods:**

We used data from the National Institutes of Health-funded Environmental influences on Child Health Outcomes (ECHO) Cohort (*n* = 1,198) that were collected from 2008 to 2019 at several sites across the United States. The present analysis was conducted in 2023–2024. Using single-pollutant and mixture models, we examined associations between prenatal phenol concentrations and three key child sleep quality outcomes: sleep problems, disturbance, and impairment. Child sleep outcomes were assessed using the Child Behavior Checklist (CBCL) and the Patient-Reported Outcomes Measurement Information System (PROMIS) Sleep Disturbance and Sleep-Related Impairment scales. Unadjusted and multivariable-adjusted models were examined, with stratified models and interaction terms used to examine interactions with child sex.

**Results:**

Of the eight phenols assessed, higher prenatal methylparaben concentrations were associated with lower child sleep-related impairment scores (*β* = −4.79, 95% CI: −9.45 to −0.14). Sex modified the associations for benzophenone-3 and PROMIS sleep disturbance T-scores, where the association was stronger among boys (tertile 3 vs. 1, *β* = 3.20; 95% CI: 0.27–6.14; *p* = 0.033) and did not persist among girls. Bisphenol A was associated with sleep-related impairment among boys (tertile 2 vs. 1, *β* = −5.69; 95% CI: 0.55–10.82; *p* = 0.031). Phenol mixtures were not associated with sleep outcomes overall or by sex.

**Conclusion:**

The findings suggest that phenol exposure during pregnancy may be associated with child sleep quality and that child sex modifies this association.

## 1 Introduction

Childhood sleep quality is an understudied public health concern, but existing research has shown that it can predict continued sleep quality issues and other health outcomes throughout life. Epidemiologic studies identify child sleep problems, such as insufficient and disrupted sleep, to be common and widespread (1–8), impacting an estimated 20%–50% of infants and school-age children (9–11). According to a report by the U.S. Centers for Disease Control and Prevention (CDC), 1 in every 3 children from early infancy through adolescence suffers from insufficient sleep, disproportionately affecting racial and ethnic minorities with lower socioeconomic status and special health care needs (12).

Sleep is vital for healthy development in childhood, as poor sleep (in quantity and/or quality) can have detrimental physical, neurocognitive, emotional, and behavioral impacts (8, 9, 13–18). Sleep disruption in adolescents negatively impacts school performance (1), mental health, and risk-taking behaviors (9, 13, 19). Sleep disturbances in childhood and adolescence can precede sleep disorders in adulthood, affecting overall health and costing hundreds of billions of dollars in the United States annually on sleep healthcare and pharmaceutical intervention (13). Risk factors for poor sleep quality in children include genetics, nutrition, the caregiver-child relationship, and community/socioeconomic (1, 12) and environmental (20) factors (10, 21). Prenatal factors, such as maternal alcohol consumption (10, 22–24), maternal depression (25), and maternal sleep duration (26, 27) can also play a role in childhood sleep quality.

A growing body of literature implicates *in utero* exposure to endocrine-disrupting chemicals (EDCs) as having negative effects on childhood neurobehavioral development (28–42), which may manifest in both behavioral and sleep disturbances (43–45). Endocrine-disrupting chemicals such as phenols present in common household personal care and beauty products have been found to be prevalent in the bodies of pregnant women in the United States (46–51).

While previous studies implicate chemical exposure as affecting adult (52–56) and adolescent (57) sleep quality, existing literature examining whether chemical exposure during pregnancy may affect offspring sleep quality is scarce. Phenol exposure *in utero* has been shown to alter brain development; the endocrine-disrupting properties of phenols affect brain masculinization, having potential implications for a variety of neurodevelopmental outcomes, including child circadian rhythms, potentially through disruption of the suprachiasmatic nucleus or hormone-regulated sleep pathways (58–62).

Several epidemiologic studies offer early-life insight into this relationship. Zamora et al. found positive associations between maternal prenatal exposure to the pesticide chlorpyrifos and later sleep timing among adolescent offspring, wherein the highest tertile of exposure was associated with a 0.6-hour later sleep midpoint [95% confidence interval (CI): 0.01–1.3; *p*-trend = 0.01] (45). Previous studies also shed light on differential sex impacts of prenatal chemical concentrations. For instance, Geiger et al. reported that sex modified the relationship between bisphenol A (BPA) exposure and scores on the Child Behavior Checklist (CBCL) sleep problems syndrome sale, where the association was inverse among males with lower BPA concentrations and positive (more reported behavior problems) among girls in the higher BPA group (60). However, these studies primarily examined general behavioral outcomes or sleep timing rather than validated measures of sleep quality including sleep-related impairment.

The literature on prenatal phenol exposure and sleep outcomes among children is limited, and findings are inconsistent (60, 63, 64). For instance, Geiger et al. (60) evaluated sleep problems using the CBCL but did use not instruments exclusively designed for sleep quality assessment. Two other studies have explored prenatal phenol exposure in relation to behavior or circadian timing (63, 64) but not using validated sleep outcomes. To our knowledge, no prior studies have directly examined the association between prenatal phenol exposure and sleep quality outcomes in early childhood using CBCL or Patient-Reported Outcomes Measurement Information System (PROMIS) assessments. Additionally, no studies have evaluated prenatal chemical exposure in relation to PROMIS sleep disturbance or impairment outcomes. These gaps underscore the need for research leveraging standardized sleep assessments.

Our study addresses the knowledge gap by examining the association between prenatal environmental phenol exposure and offspring childhood sleep quality, which can interact and amplify health effects, using single-pollutant and mixture models (48). We hypothesized that higher maternal phenol exposure during pregnancy would be associated with more offspring sleep problems, disturbances, and related daytime impairments. We explored this hypothesis using data from a large, racially/ethnically, geographically, and socioeconomically diverse sample of children across the United States who are part of the National Institutes of Health-funded Environmental influences on Child Health Outcomes (ECHO) Cohort, a nationwide consortium of pediatric cohorts assembled with the aim of leveraging demographic and geographic heterogeneity and a large sample size to address research questions pertaining to children's environmental health (65, 66).

## 2 Materials and methods

### 2.1 Study population

Mother-child dyads from the ECHO Cohort meeting the following criteria were included: (1) phenols were measured in maternal urine at least once during pregnancy; (2) the child had complete data for the preschool CBCL sleep syndrome scale (for ages 1.5–5 years) (67), the PROMIS Parent Proxy Short Form-Sleep Disturbance 4a (PSD4a) measure (68, 69), or the PROMIS Parent Proxy Short Form Sleep-related Impairment 4a measure (PSRI4a) (70, 71) at least once between the ages of 4 and 8 years; and (3) the pregnancy was a singleton gestation. For mothers with multiple births, the firstborn child was selected. Supplementary Table S1 details cohorts and sample sizes by child sleep outcome. Supplementary Figure S1 illustrates how analytical samples were derived. A total of 1,198 children contributed data to 1 or more of the 3 outcome measures.

### 2.2 Environmental phenols

The laboratory methods used to measure phenolic compounds varied across cohorts (Supplementary Table S2). Phenols were measured in maternal urine [in nanograms (ng) per milliliter (ml)] at 3 laboratories: the California Department of Toxic Substances Control, the CDC, and the Wadsworth Human Health Exposure Analysis Resource Laboratory (72). We used urine as the single matrix of chemical measurement because the collection of urine biomarkers is recognized as “the most common, reliable, and non-invasive method used to measure BPA” (73). The number of phenols measured in each cohort ranged from 8 to 14. Phenols were included in the analysis 75% or more of the values were above the method limit of detection (LOD). Detection rates for these compounds are presented in Table 1. Eight phenols met these criteria: BPA, BPS, benzophenone-3 (BP3), 2,4-dichlorophenol (DCP24), 2,5-dichlorophenol (DCP25), methylparaben, and propylparaben. For those observations below the LOD, we imputed exposure values as the LOD divided by the square root of 2 (74, 75). Phenol concentrations were adjusted for urinary dilution using the specific gravity (SG), or creatinine when the SG was not available: {SG_adj = phenol value*(SG_median-1)/(SG-1); creatinine_adj = phenol value*creatinine median/creatinine}. Extreme phenol values over the 99th percentile were excluded (76). For cohorts with phenols measured at multiple gestational time points, concentrations above the LOD were averaged.

**Table 1:** Detection rate and distribution of urinary phenols (ng/ml).

Chemical	CBCL outcome		PSD4a outcome		PSRI4a outcome	
	Detection rate *N* (%)	Median (Range)	Detection rate *N* (%)	Median (Range)	Detection rate *N* (%)	Median (Range)
Benzophenone-3	650 (99)	84.68 (0.05–5,832.60)	368 (99)	75.85 (2.05–5,832.60)	141 (100)	66.84 (2.05–4,862.78)
Bisphenol A	899 (88)	1.02 (0.04–10.55)	344 (93)	1.12 (0.12–13.04)	139 (99)	1.65 (0.30–13.04)
Bisphenol S	800 (87)	0.49 (0.02–9.32)	270 (93)	0.57 (0.05–8.08)	125 (91)	0.48 (0.05–4.87)
2,4-Dichlorophenol	467 (97)	0.66 (0.08–58.37)	165 (98)	0.94 (0.10–52.72)	139 (98)	0.98 (0.10–52.72)
2,5-Dichlorophenol	467 (97)	2.11 (0.06–1,612.68)	167 (100)	6.71 (0.24–1,612.68)	141 (100)	7.13 (0.48–709.25)
Methylparaben	592 (100)	89.51 (1.43–1,442.89)	164 (99)	90.69 (2.45–1,211.27)	138 (99)	96.42 (2.45–1,166.51)
Propylparaben	589 (100)	15.62 (0.16–569.30)	166 (99)	15.26 (0.22–559.87)	140 (99)	15.0 (0.26–559.87)
Triclosan	661 (90)	11.67 (0.25–1,049.13)	315 (86)	12.30 (0.40–956.42)	118 (85)	20.10 (1.11–956.42)

CBCL, child behavior checklist; PSD4a, PROMIS parent proxy short form-sleep disturbance 4a; PSRI4a, PROMIS parent proxy short form sleep-related impairment 4a.

Values <limit of detection (LOD) were imputed as LOD/sqrt(2). Values were adjusted for specific gravity or creatinine, whichever was available.

### 2.3 Sleep

Three sleep outcome measures were used to examine separate aspects of sleep quality: sleep problems, sleep disturbance, and sleep-related impairment.

Sleep problems were assessed with the Preschool CBCL, a validated and commonly used parent-report of child behavior for children aged 1.5–5 years (77, 78). The CBCL includes seven items on a “syndrome scale” for measuring sleep problems: “does not want to sleep alone”; “has nightmares”; “has trouble getting to sleep”; “resists going to bed”; “sleeps less than most children”; “talks during sleep”; and “wakes often”. Scores on the sleep syndrome scale are calculated to determine how well the item describes the child's sleep in the past 2 months using a 3-point scale. Higher scores correspond to more sleep problems, where “not true” = 0 points, “somewhat true” = 1 point, and “often true” = 2 points. CBCL sleep syndrome scores were converted to T-scores according to the CBCL manual for the study analysis.

Sleep disturbance was evaluated with the PSD4a for children aged 2 to <8 years [71]. On a 5-point scale (“never”, “almost never”, “sometimes”, “almost always”, and “always”), parents report how often in the past 7 days their child had difficulty falling asleep, trouble sleeping through the night, a problem with sleep, and trouble sleeping. We used the PROMIS T-score with a mean of 50 and a standard deviation (SD) of 10.

Sleep-related impairment was measured using the PSRI4a for 5 to <8-year-olds. Sleep impairments are distinct from sleep disturbances in that they are decrements in functioning during waking hours due to sleep disturbances or other sleep quality issues (70). On a 5-point scale (“never”, “almost never”, “sometimes”, “almost always”, and “always”), parents report how frequently over the past 7 days their child was sleepy during the daytime, had trouble concentrating, had difficulty completing tasks due to sleepiness, and had problems during the day due to poor sleep. We used the PROMIS T-score as described above.

If any of the three sleep assessments was completed more than once for a child aged 4–<8 years, the average score was used to provide a smoothed assessment of sleep quality during early childhood as opposed to a worst-case scenario assessment (79).

### 2.4 Covariates

Covariates included in this analysis were child age in years (at outcome assessment), child gestational age at birth, highest level of maternal education (<high school, high school or equivalent, and some college or more), maternal age at delivery, and cohort. These variables were selected based on their relevance in previous epidemiologic studies of prenatal exposures and child sleep, as well as theoretical causal pathways. We examined correlations between each potential confounder and the key exposure and outcome variables, which, together with the existing literature, informed our models. We removed birth weight from the models since it was moderately correlated with gestational age. Child sex was reported by the parent/caregiver and dichotomized as male vs. female. Child gestational age at birth (in weeks) was harmonized from multiple sources (medical record abstraction or self-report). Child race and Hispanic ethnicity were reported by the parent/caregiver, and categories across cohorts were harmonized as Non-Hispanic White, Non-Hispanic Black, Non-Hispanic Other, and Hispanic to maximize the sample size within categories (80). Covariates with missing data were imputed using multiple imputation by chained equations, treating cohort as a cluster variable with 10 imputed datasets and 5 iterations for each imputed dataset (80).

### 2.5 Analysis

#### 2.5.1 Single pollutant models

We evaluated each sleep outcome using separate models due to differing available data samples. For each outcome, analysis of variance assessed differences by sex, education, race, and cohort. For example, we tested whether there was a significant difference in sleep problems (CBCL assessment) between female and male children. We used unadjusted and adjusted linear mixed effect regression models to estimate the association of prenatal maternal phenol categories of increasing exposure (see Supplementary Table S3 for ranges of tertile values) with child sleep outcomes. We chose tertiles rather than linear or spline terms due to the highly skewed distribution of most phenol concentrations, and to explore a dose-response relationship. Visual inspection indicated that categorical treatment improved robustness and interpretability. In the adjusted models, we included maternal age at delivery, maternal education, child sex, child gestational age, and child age at the sleep outcome as covariates, with random intercepts for cohorts to account for within-cohort correlation. Where interaction terms (e.g., sex*exposure) were evaluated, we confirmed that main effects remained stable compared to models excluding interactions. We also conducted a sensitivity analysis for gestational age at birth by running the multivariable-adjusted model without this variable.

#### 2.5.2 Sex-stratified analyses

To evaluate potential effect modification by child sex, we included multiplicative interaction terms between phenol exposure tertiles and child sex (male vs. female) within our linear mixed-effects models. Each model included cross-product terms representing the interaction between exposure category and sex, while accounting for covariates and random intercepts by cohort. Statistical significance of interaction terms was assessed using a *p*-value threshold of <0.10, which is commonly used in epidemiologic research to identify potential effect modification without overly conservative filtering. This approach allowed us to examine whether the association between prenatal phenol exposure and sleep outcomes differed meaningfully by child sex. While we did not estimate additive interaction (e.g., using risk differences or relative excess risk due to interaction), we recognize that such approaches provide important complementary information on public health impact. Although not implemented here, future work could apply additive interaction frameworks to more comprehensively assess sex-specific vulnerability.

#### 2.5.3 Mixture models

Quantile g-computation (81) was used to model combined exposure to all 8 phenols considered in this analysis and each of the 3 aspects of sleep quality at ages 4–<8. In the quantile g-computation, parametric, generalized linear model-based computation was performed to estimate the effect of increasing all phenols by 1 quartile on each of the 3 sleep outcomes. Each exposure was assigned a positive or negative weight depending on the direction of its association with the outcome. The weights for each outcome, potentially both positive and negative, summed to 1, representing the proportion of total effect contributed by the individual partial effects (positive or negative) of each phenol chemical. All analyses were conducted using R Statistical Software (version 4.1.2, R Core Team, Vienna, Austria), and the gcomp R package (version 2.9.0, R Core Team, Vienna, Austria) was used for the mixture analysis (81, 82).

## 3 Results

### 3.1 Participant characteristics

Of the 1,198 total participants included in our analyses, the CBCL sleep subsample was the largest (1,123), followed by the PSD4a (374) and PSRI4a (352) subsamples (Table 2). Overlap existed between analytical groups, which were based on the three sleep quality outcomes. Overlaps were *N* = 299 between the CBCL Sleep Problems and PSD4a subsamples, *N* = 107 between the Sleep Problems and PSRI subsamples, and *N* = 142 between the PSD4a and PSRI4a subsamples.

**Table 2:** Descriptive characteristics of the study sample by sleep quality outcome.

Characteristic	CBCL		PSD4a		PSRI4a	
	*N* = 1,123	*p*-value^a^	*N* = 374	*p*-value^a^	*N* = 142	*p*-value^a^
Child age (years)		0.7		0.08		0.8
Mean (SD)	4.2 (0.4)		4.9 (0.8)		5.4 (1.0)	
Median (IQR)	4.0 (4.0, 4.5)		4.7 (4.4, 5.1)		5.1 (4.9, 5.9)	
Missing	0		0		0	
Child sex, *N* (%)		0.7		0.6		0.4
Male	580 (52%)		185 (49%)		66 (46%)	
Female	543 (48%		189 (51%)		76 (54%)	
Child race/ethnicity, *N* (%)		0.7		0.8		0.3
Non-Hispanic White	385 (35%)		51 (14%)		23 (16%)	
Non-Hispanic Black	91 (8.3%)		<10 (<5%)		<5 (<5%)	
Non-Hispanic Asian	61 (5.5%)		44 (12%)		<5 (<5%)	
Non-Hispanic other	90 (8.2%)		36 (9.7%)		8 (5.6%)	
Hispanic	476 (43%)		235 (63%)		110 (77%)	
Missing	20		<5		0	
Maternal age (years)		0.3		0.7		0.8
Mean (SD)	30.8 (5.5)		31.4 (4.9)		30.1 (5.1)	
Median (IQR)	31.0 (27.0, 35.0)		32.0 (28.0, 35.0)		30.0 (27.0, 34.0)	
Missing	0		0		0	
Gestational age at birth (months)		0.7		0.9		>0.9
Mean (SD)	38.8 (1.7)		38.5 (2.0)		38.2 (2.1)	
Median (IQR)	39.0 (38.0, 40.0)		39.0 (38.0, 40.0)		39.0 (38.0, 39.0)	
Missing	0		0		0	
Birth weight (grams)		<0.001		0.009		0.02
Mean (SD)	3,313.3 (518.7)		3,293.9 (523.1)		3,227.2 (575.5)	
Median (IQR)	3,340.0 (2,990.0, 3,651.2)		3,340.0 (2,977.8, 3,621.8)		3,232.0 (2,941.0, 3,607.2)	
Missing	31		8		6	
Maternal education, *N* (%)		0.5		0.4		0.4
Less than high school	88 (7.9%)		<5 (<5%)		<5 (<5%)	
High school degree, GED/equivalent	178 (16%)		31 (8.3%)		11 (7.8%)	
Some college, no degree, and above	848 (76%)		339 (91%)		129 (91%)	
Missing	9		2		1	
Annual household income, *N* (%)		0.14		0.2		0.4
<$30,000	219 (32%)		95 (29%)		65 (50%)	
$30,000–$49,999	82 (12%)		56 (17%)		27 (21%)	
$50,000–$74,999	84 (12%)		45 (14%)		16 (12%)	
$75,000 or more	293 (43%)		136 (41%)		23 (18%)	
Missing	445		42		221	
Pre-pregnancy BMI category, *N* (%)		0.6		0.3		0.2
Underweight	27 (2.6%)		10 (2.7%)		6 (4.4%)	
Normal weight	454 (44%)		148 (40%)		61 (45%)	
Overweight	286 (28%)		96 (26%)		36 (26%)	
Obese	267 (26%)		114 (31%)		34 (25%)	
Missing	89		6		5	
Sleep Quality		0.91		0.03		0.03
Mean (SD)	53.08 (5.36)		50.66 (9.07)		47.55 (8.87)	
Median (IQR)	51 (50.0, 53.0)		49.64 (41.39, 57.14)		46.22 (39.95, 51.49)	

BMI, body mass index; CBCL, child behavior checklist; GED, general educational development; IQR, interquartile range; PSD4a, PROMIS parent proxy short form-sleep disturbance 4a; PSRI4a, PROMIS parent proxy short form sleep-related impairment 4a; SD, standard deviation.

^a^ Welch's two-sample *t*-test and Pearson's chi-squared test for sex difference for continuous and categorical variables.

Child age ranged from a mean of 4.2 (CBCL group) to 6.7 (PSRI4a group) years, and child ethnicity was predominantly Hispanic, followed by Non-Hispanic White. Mothers were overall highly educated, with at least some college or above, and predominantly did not have obesity pre-pregnancy. Sleep outcome scores (sleep problems, sleep disturbance, and sleep-related impairment) did not significantly differ by sociodemographic characteristics.

### 3.2 Single pollutant models

In analyzing maternal urinary phenols, the following chemicals had a greater than 75% detection rate across the three sleep quality outcome measure subsamples for children with available data: BP3, BPA, BPS, DCP24, DCP25, methylparaben, propylparaben, and triclosan (Table 1).

Both methylparaben and ethylparaben were detected in 99%–100% of pregnant people across the subsamples, while BPA and BPS were somewhat lower in prevalence. BPS had the lowest concentrations in the urine of mothers of children with sleep problems (CBCL: 0.49 ng/ml), sleep disturbance (PSD4a: 0.57 ng/ml), and sleep impairment (PSRI4a: 0.48 ng/ml). By contrast, methylparaben had the highest concentrations, at 89.51 ng/ml, 90.69 ng/ml, and 96.42 ng/ml, respectively, followed most closely by BP3. However, BP3 had a larger median range between the sleep outcomes (highest for sleep problems, at 84.68 ng/ml, and lowest for sleep impairment, at 66.84 ng/ml). DCP25 had a lower concentration but had the largest median range across the sleep outcomes, from 2.11 ng/ml for sleep problems to 7.13 ng/ml for sleep impairment.

We assessed direct associations of eight environmental phenols with child sleep quality and their interactions with child sex by including interaction terms in the regression models (Table 3). The direction of the association between most of the chemicals and sleep quality was positive, meaning that higher phenol concentrations were associated with worse sleep outcomes. For BP3, the strength of the association increased across tertiles in the CBCL and PSD4a samples, suggesting a dose-response trend. For the PSRI4a outcome, the estimate was slightly higher in tertile 2 (*β* = 2.80; 95% CI: −2.13–7.73) than in tertile 3 (*β* = 2.34; 95% CI: −2.42–7.10). Several chemicals were inversely related to sleep outcomes, but only methylparaben reached statistical significance, with each unit increase associated with a 4.79-point decrease in PSRI4a score (95% CI: −9.45 −0.14; *p* = 0.04).

**Table 3:** Results of linear mixed regression models assessing the association between tertiles of phenol exposure during pregnancy and child sleep outcomes.

Phenol	CBCL				PSD				PSRI4a		
Tertile (range)	Estimate	95% CI	*p*-value	Tertile (range)	Estimate	95% CI	*p*-value	Tertile (range)	Estimate	95% CI	*p*-value
BP3				BP3				BP3			
Tertile 1 (0.05, 38.12)		Referent		Tertile 1 (2.05, 32.23)		Referent		Tertile 1 (2.05, 29.28)		Referent	
Tertile 2 (38.12, 214.56)	0.39	−1.14, 1.92	0.62	Tertile 2 (32.23, 207.12)	0.58	−2.33, 3.49	0.70	Tertile 2 (29.28, 142.39)	2.80	−2.13, 7.73	0.26
Tertile 3 (214.56, 5832.60)	1.01	−0.57, 2.59	0.21	Tertile 3 (207.12, 5832.60)	2.08	−0.89, 5.05	0.17	Tertile 3 (142.39, 4862.78)	2.34	−2.42, 7.10	0.33
Tertile 2 X child sex	0.48	−1.69, 2.64	0.67	Tertile 2 X child sex	0.90	−3.24, 5.04	0.67	Tertile 2 X child sex	−4.18	−11.09, 2.73	0.23
Tertile 3 X child sex	0.07	−2.10, 2.23	0.95	Tertile 3 X child sex	0.78	−3.31, 4.88	0.71	Tertile 3 X child sex	−0.82	−7.81, 6.17	0.82
BPA				BPA				BPA			
Tertile 1 (0.04, 0.70)		Referent		Tertile 1 (0.12, 0.79)		Referent		Tertile 1 (0.29, 1.30)		Referent	
Tertile 2 (0.70, 1.44)	−0.04	−1.18, 1.10	0.94	Tertile 2 (0.79, 1.61)	−0.33	−3.22, 2.56	0.82	Tertile 2 (1.30, 2.04)	−0.97	−5.70, 3.76	0.68
Tertile 3 (1.44, 10.55)	0.56	−0.58, 1.71	0.33	Tertile 3 (0.79, 1.61)	−0.06	−3.27, 3.15	0.97	Tertile 3 (2.04, 13.04)	−0.08	−4.58, 4.43	0.97
Tertile 2 X child sex	0.13	−1.45, 1.72	0.87	Tertile 2 X child sex	−0.40	−4.47, 3.68	0.85	Tertile 2 X child sex	7.70	1.21, 14.20	**0**.**02**
Tertile 3 X child sex	0.46	−1.14, 2.05	0.57	Tertile 3 X child sex	1.85	−2.27, 5.97	0.38	Tertile 3 X child sex	4.06	−2.72, 10.84	0.24
BPS				BPS				BPS			
Tertile 1 (0.02, 0.33)		Referent		Tertile 1 (0.05, 0.41)		Referent		Tertile 1 (0.05, 0.36)		Referent	
Tertile 2 (0.33, 0.75)	−0.69	−1.84, 0.46	0.24	Tertile 2 (0.41, 0.91)	−0.60	−3.66, 2.46	0.70	Tertile 2 (0.36, 0.75)	−1.19	−6.45, 4.06	0.65
Tertile 3 (0.75, 9.32)	−0.93	−2.06, 0.21	0.11	Tertile 3 (0.91, 8.08)	−0.72	−3.73, 2.30	0.64	Tertile 3 (0.75, 4.87)	1.57	−3.02, 6.17	0.50
Tertile 2 X child sex	0.67	−0.93, 2.27	0.41	Tertile 2 X child sex	0.84	−3.60, 5.28	0.71	Tertile 2 X child sex	3.44	−3.87, 10.75	0.35
Tertile 3 X child sex	1.29	−0.32, 2.89	0.12	Tertile 3 X child sex	−0.29	−4.73, 4.14	0.90	Tertile 3 X child sex	0.36	−6.81, 7.52	0.92
DCP 24				DCP 24				DCP 24			
Tertile 1 (0.08, 0.46)		Referent		Tertile 1 (0.10, 0.63)		Referent		Tertile 1 (0.10, 0.63)		Referent	
Tertile 2 (0.46, 1.02)	−1.16	−2.88, 0.56	0.19	Tertile 2 (0.63, 1.37)	1.40	−2.57, 5.36	0.49	Tertile 2 (0.63, 1.40)	3.45	−1.10, 8.01	0.14
Tertile 3 (1.02, 58.37)	−0.31	−2.08, 1.46	0.73	Tertile 3 (1.37, 52.72)	−3.76	−7.84, 0.32	0.07	Tertile 3 (1.40, 52.72)	−3.40	−7.84, 1.04	0.13
Tertile 2 X child sex	1.62	−0.83, 4.06	0.19	Tertile 2 X child sex	0.63	−5.23, 6.50	0.83	Tertile 2 X child sex	−0.55	−7.16, 6.05	0.87
Tertile 3 X child sex	0.23	−2.21, 2.67	0.85	Tertile 3 X child sex	4.07	−1.86, 10.01	0.18	Tertile 3 X child sex	8.94	2.07, 15.82	**0**.**01**
DCP 25				DCP 25				DCP 25			
Tertile 1 (0.06, 1.12)		Referent		Tertile 1 (0.24, 3.97)		Referent		Tertile 1 (0.48, 4.81)		Referent	
Tertile 2 (1.12, 4.46)	0.97	−0.80, 2.73	0.28	Tertile 2 (3.97, 15.27)	1.20	−3.20, 5.61	0.59	Tertile 2 (4.81, 15.72)	0.67	−4.44, 5.79	0.80
Tertile 3 (4.46, 1612.68)	−0.82	−2.61, 0.97	0.37	Tertile 3 (15.27, 1612.68)	−4.45	−9.21, 0.31	0.07	Tertile 3 (15.72, 709.25)	−3.42	−8.69, 1.84	0.20
Tertile 2 X child sex	−0.60	−3.06, 1.85	0.63	Tertile 2 X child sex	1.36	−4.59, 7.31	0.65	Tertile 2 X child sex	1.82	−5.09, 8.73	0.60
Tertile 3 X child sex	0.39	−2.08, 2.86	0.75	Tertile 3 X child sex	4.31	−1.61, 10.22	0.15	Tertile 3 X child sex	10.21	3.36, 17.06	**0**.**00**
Methylparaben				Methylparaben				Methylparaben			
Tertile 1 (1.43, 42.71)		Referent		Tertile 1 (2.45, 43.56)		Referent		Tertile 1 (2.45, 41.26)		Referent	
Tertile 2 (42.71, 171.27)	−0.81	−2.41, 0.79	0.32	Tertile 2 (43.56, 183.16)	−3.37	−7.50, 0.76	0.11	Tertile 2 (41.26, 182.83)	−4.79	−9.45, −0.14	0.04
Tertile 3 (171.27, 1442.89)	−0.02	−1.66, 1.63	0.99	Tertile 3 (183.16, 1211.27)	−0.54	−4.86, 3.77	0.80	Tertile 3 (182.83, 1166.51)	−3.80	−8.73, 1.13	0.13
Tertile 2 X child sex	0.73	−1.51, 2.97	0.52	Tertile 2 X child sex	2.71	−3.41, 8.82	0.38	Tertile 2 X child sex	1.55	−5.51, 8.62	0.66
Tertile 3 X child sex	0.45	−1.80, 2.69	0.70	Tertile 3 X child sex	0.72	−5.27, 6.70	0.81	Tertile 3 X child sex	4.59	−2.37, 11.55	0.19
Propylparaben				Propylparaben				Propylparaben			
Tertile 1 (0.16, 6.31)		Referent		Tertile 1 (0.22, 5.69)		Referent		Tertile 1 (0.26, 5.33)		Referent	
Tertile 2 (6.31, 42.98)	−0.95	−2.51, 0.61	0.23	Tertile 2 (5.69, 36.69)	0.25	−3.80, 4.31	0.90	Tertile 2 (5.33, 34.61)	1.04	−3.67, 5.74	0.66
Tertile 3 (42.98, 569.30)	−0.27	−1.85, 1.31	0.74	Tertile 3 (36.69, 559.87)	1.46	−2.87, 5.80	0.51	Tertile 3 (34.61, 559.87)	−2.55	−7.44, 2.35	0.30
Tertile 2 X child sex	−2.12	−4.33, 0.09	**0**.**06**	Tertile 2 X child sex	1.33	−4.92, 7.58	0.68	Tertile 2 X child sex	−0.36	−7.53, 6.81	0.92
Tertile 3 X child sex	−1.52	−3.73, 0.68	0.17	Tertile 3 X child sex	−1.42	−7.45, 4.62	0.64	Tertile 3 X child sex	5.17	−1.88, 12.22	0.15
Triclosan				Triclosan				Triclosan			
Tertile 1 (0.24, 5.76)		Referent		Tertile 1 (0.40, 5.07)		Referent		Tertile 1 (1.11, 5.71)		Referent	
Tertile2 (5.76, 32.60)	−0.03	−1.53, 1.46	0.97	Tertile2 (5.07, 62.26)	−0.39	−3.43, 2.64	0.80	Tertile2 (5.71, 101.86)	1.71	−3.32, 6.75	0.50
Tertile3 (32.60, 1049.13)	0.01	−1.44, 1.46	0.99	Tertile3 (62.26, 956.42)	−0.27	−3.13, 2.59	0.85	Tertile3 (101.86, 956.42)	−2.69	−7.51, 2.14	0.27
Tertile 2 X child sex	0.65	−1.37, 2.68	0.53	Tertile 2 X child sex	1.25	−2.86, 5.36	0.55	Tertile 2 X child sex	−4.42	−11.43, 2.58	0.21
Tertile 3 X child sex	0.11	−1.92, 2.13	0.92	Tertile 3 X child sex	1.24	−2.91, 5.40	0.56	Tertile 3 X child sex	0.65	−6.48, 7.77	0.86

BP3, benzophenone-3; BPA, bisphenol A; BPS, bisphenol S; CBCL, Child Behavior Checklist; CI, confidence interval; DCP24, 2,4-dichlorophenol; DCP25, 2,5-dichlorophenol; PSD4a, PROMIS Parent Proxy Short Form-Sleep Disturbance 4a; PSRI4a, PROMIS Parent Proxy Short Form Sleep-related Impairment 4a.

Model adjusted for child age in years (at outcome assessment), child gestational age at birth, highest level of maternal education (<high school, high school or equivalent, and some college or more), and maternal age at delivery.

Bold text indicates significance at the *p*<0.10 level.

Higher scores on sleep measures indicate more sleep issues.

### 3.3 Sex-stratified analyses

Chemical × sex interaction terms were significant for BPA, DCP24, DCP25, and PRPB (Table 3). The main effects in Table 3 are from models that include interaction terms. Sensitivity checks showed consistent beta estimates when excluding interaction terms. The sensitivity analysis for gestational age at birth yielded results that were very similar with and without the variable included in the model. Since the multiplicative interaction terms in Table 3 suggested sex to be an effect modifier, we then assessed the association between tertiles of phenol exposure during pregnancy and child sleep outcomes by child sex (Table 4). Exposure tertile ranges are now included in Table 4. There were two statistically significant associations among boys, but these associations were null among their female counterparts. Specifically, boys in the highest category of BP3 concentration had 3.20-point higher PSD4a scores on average compared to their counterparts in the referent category (95% CI: 0.27–6.14), and those in BPA tertile 2 had higher PSRI4a scores than those in the referent category (*β* = 5.69; 95% CI: 0.55–10.82). Among the relationships that were not statistically significant, the direction and strength of associations were mixed.

**Table 4:** Results of linear mixed regression models assessing the association between tertiles of phenol exposure during pregnancy and child sleep outcomes by child sex.

CBCL				
Phenol	Sample size (boys/girls)	Urinary phenol level (Cutoff)	Boys β (95% CI)	Girls β (95% CI)
BP3	331/324	Tertile 1 (0.05, 41.76)	0 (ref)	0 (ref)
		Tertile 2 (41.76, 246.12)	0.75 (−0.85, 2.35)	0.48 (−1.03, 2.00)
		Tertile 3 (246.12, 4769.08)	0.92 (−0.66, 2.50)	1.21 (−0.37, 2.78)
BPA	525/502	Tertile 1 (0.04, 0.70)	0 (ref)	0 (ref)
		Tertile 2 (0.70, 1.42)	0.11 (−1.01, 1.22)	0.09 (−1.06, 1.24)
		Tertile 3 (1.42, 10.55)	0.99 (−0.16, 2.15)	0.66 (−0.50, 1.82)
BPS	463/456	Tertile 1 (0.04, 0.34)	0 (ref)	0 (ref)
		Tertile 2 (0.34, 0.74)	−0.16 (−1.28, 0.97)	−0.67 (−1.85, 0.52)
		Tertile 3 (0.74, 9.16)	0.26 (−0.91, 1.43)	−1.04 (−2.22, 0.14)
DCP24	238/244	Tertile 1 (0.13, 0.45)	0 (ref)	0 (ref)
		Tertile 2 (0.45, 1.06)	0.24 (−1.48, 1.96)	−1.06 (−2.83, 0.72)
		Tertile 3 (1.06, 45.78)	−0.35 (−2.04, 1.35)	−0.32 (−2.13, 1.49)
DCP25	238/241	Tertile 1 (0.15, 0.99)	0 (ref)	0 (ref)
		Tertile 2 (0.99, 3.98)	0.55 (−1.13, 2.23)	0.91 (−0.92, 2.73)
		Tertile 3 (3.98, 1612.68)	−0.31 (−2.13, 1.51)	−0.73 (−2.60, 1.14)
Methylparaben	302/289	Tertile 1 (1.43, 41.33)	0 (ref)	0 (ref)
		Tertile 2 (41.33, 175.61)	−0.03 (−1.61, 1.56)	−0.95 (−2.56, 0.66)
		Tertile 3 (175.61, 1176.74)	0.50 (−1.06, 2.06)	−0.06 (−1.68, 1.56)
Propylparaben	299/290	Tertile 1 (0.22, 6.96)	0 (ref)	0 (ref)
		Tertile 2 (6.96, 44.08)	1.10 (−0.48, 2.67)	−0.89 (−2.44, 0.67)
		Tertile 3 (44.08, 513.55)	1.31 (−0.27, 2.90)	−0.31 (−1.88, 1.25)
Triclosan	377/358	Tertile 1 (0.24, 5.45)	0 (ref)	0 (ref)
		Tertile 2 (5.45, 28.13)	0.71 (−0.72, 2.14)	0.06 (−1.40, 1.52)
		Tertile 3 (28.13, 842.12)	0.03 (−1.45, 1.51)	0.05 (−1.37, 1.47)
PSD4a				
Phenol	Sample size (boys/girls)	Urinary phenol level (Cutoff)	Boys β (95% CI)	Girls β (95% CI)
BP3	183/187	Tertile 1 (2.61, 31.49)	0 (ref)	0 (ref)
		Tertile 2 (31.49, 227.19)	1.77 (−1.25, 4.79)	−0.04 (−3.08, 2.99)
		Tertile 3 (227.19, 4769.08)	**3.20 (0.27, 6.14) **	1.81 (−1.26, 4.88)
BPA	183/187	Tertile 1 (0.18, 0.83)	0 (ref)	0 (ref)
		Tertile 2 (0.83, 1.70)	−0.69 (−3.75, 2.36)	−0.60 (−3.61, 2.41)
		Tertile 3 (1.70, 8.83)	1.97 (−1.27, 5.20)	−0.74 (−4.32, 2.84)
BPS	136/155	Tertile 1 (0.09, 0.42)	0 (ref)	0 (ref)
		Tertile 2 (0.42, 0.91)	−0.21 (−3.50, 3.09)	−0.26 (−3.45, 2.94)
		Tertile 3 (0.91, 8.08)	−1.35 (−4.70, 2.01)	−0.35 (−3.51, 2.81)
DCP24	79/89	Tertile 1 (0.14, 0.63)	0 (ref)	0 (ref)
		Tertile 2 (0.63, 1.49)	2.57 (−1.70, 6.84)	1.21 (−3.11, 5.53)
		Tertile 3 (1.49, 45.78)	−0.27 (−4.60, 4.05)	−3.78 (−8.24, 0.68)
DCP25	79/88	Tertile 1 (0.24, 3.16)	0 (ref)	0 (ref)
		Tertile 2 (3.16, 17.88)	1.22 (−4.41, 6.86)	1.34 (−3.88, 6.56)
		Tertile 3 (17.88, 1612.68)	−1.88 (−7.48, 3.71)	−4.36 (−10.05, 1.33)
Methylparaben	78/87	Tertile 1 (2.45, 45.15)	0 (ref)	0 (ref)
		Tertile 2 (45.15, 219.97)	−0.56 (−4.88, 3.76)	−3.12 (−7.76, 1.53)
		Tertile 3 (219.97, 1211.27)	−0.21 (−4.32, 3.90)	−0.30 (−5.12, 4.51)
Propylparaben	78/89	Tertile 1 (0.22, 8.55)	0 (ref)	0 (ref)
		Tertile 2 (8.55, 47.21)	2.22 (−2.24, 6.67)	0.74 (−3.78, 5.27)
		Tertile 3 (47.21, 513.55)	−0.24 (−4.34, 3.86)	1.68 (−3.15, 6.51)
Triclosan	183/185	Tertile 1 (0.72, 5.12)	0 (ref)	0 (ref)
		Tertile 2 (5.12, 39.37)	1.24 (−1.59, 4.08)	−0.22 (−3.40, 2.96)
		Tertile 3 (39.37, 842.12)	1.21 (−1.77, 4.20)	−0.33 (−3.31, 2.65)
PSRI4a				
Phenol	Sample size (boys/girls)	Urinary phenol level (Cutoff)	Boys β (95% CI)	Girls β (95% CI)
BP3	66/75	Tertile 1 (4.29, 29.30)	0 (ref)	0 (ref)
		Tertile 2 (29.30, 104.57)	−0.38 (−5.34, 4.59)	3.01 (−2.12, 8.14)
		Tertile 3 (104.57, 4769.08)	1.27 (−4.02, 6.56)	2.63 (−2.41, 7.67)
BPA	66/74	Tertile 1 (0.35, 1.33)	0 (ref)	0 (ref)
		Tertile 2 (1.33, 1.96)	**5.69 (−0.55, 10.82) **	−0.61 (−5.57, 4.36)
		Tertile 3 (1.96, 8.83)	2.85 (−2.82, 8.51)	0.18 (−4.62, 4.98)
BPS	64/74	Tertile 1 (0.09, 0.38)	0 (ref)	0 (ref)
		Tertile 2 (0.38, 0.66)	1.95 (−3.15, 7.05)	−0.77 (−6.20, 4.66)
		Tertile 3 (0.66, 2.19)	2.27 (−3.35, 7.88)	1.77 (−2.93, 6.47)
DCP24	66/76	Tertile 1 (0.14, 0.64)	0 (ref)	0 (ref)
		Tertile 2 (0.64, 1.37)	1.78 (−3.41, 6.97)	3.88 (−0.76, 8.53)
		Tertile 3 (1.37, 17.61)	5.15 (−1.63, 10.94)	−2.92 (−7.46, 1.63)
DCP25	66/75	Tertile 1 (0.57, 4.87)	0 (ref)	0 (ref)
		Tertile 2 (4.87, 16.00)	1.85 (−4.87, 8.57)	0.91 (−4.82, 6.65)
		Tertile 3 (16.00, 709.25)	**6.45 (−0.16, 13.06) **	−2.83 (−8.78, 3.12)
Methylparaben	65/74	Tertile 1 (2.45, 44.31)	0 (ref)	0 (ref)
		Tertile 2 (44.31, 206.99)	**−**4.61** (−10.01, 0.79) **	−3.91 (−8.92, 1.10)
		Tertile 3 (206.99, 1014.06)	−0.73 (−5.72, 4.26)	−2.55 (−7.88, 2.77)
Propylparaben	65/76	Tertile 1 (0.38, 8.53)	0 (ref)	0 (ref)
		Tertile 2 (8.53, 38.41)	0.11 (−5.33, 5.55)	1.44 (−3.50, 6.38)
		Tertile 3 (38.41, 513.55)	1.36 (−3.88, 6.61)	−1.32 (−6.54, 3.89)
Triclosan	65/74	Tertile 1 (1.11, 4.99)	0 (ref)	0 (ref)
		Tertile 2 (4.99, 40.36)	−2.67 (−7.64, 2.30)	1.27 (−4.07, 6.61)
		Tertile 3 (40.36, 636.41)	−3.41 (−8.87, 2.06)	−2.25 (−7.36, 2.85)

BP3, benzophenone-3; BPA, bisphenol A; BPS, bisphenol S; CBCL, Child Behavior Checklist; CI, confidence interval; DCP24, 2,4-dichlorophenol; DCP25, 2,5-dichlorophenol; PSD4a, PROMIS Parent Proxy Short Form-Sleep Disturbance 4a; PSRI4a, PROMIS Parent Proxy Short Form Sleep-related Impairment 4a.

Model adjusted for child age in years (at outcome assessment), child gestational age at birth, highest level of maternal education (<high school, high school or equivalent, and some college or more), and maternal age at delivery.

Bolding indicates significance at the *p*<0.05 level.

Higher scores on sleep measures indicate more sleep issues.

### 3.4 Mixture models

**Figure 1:**
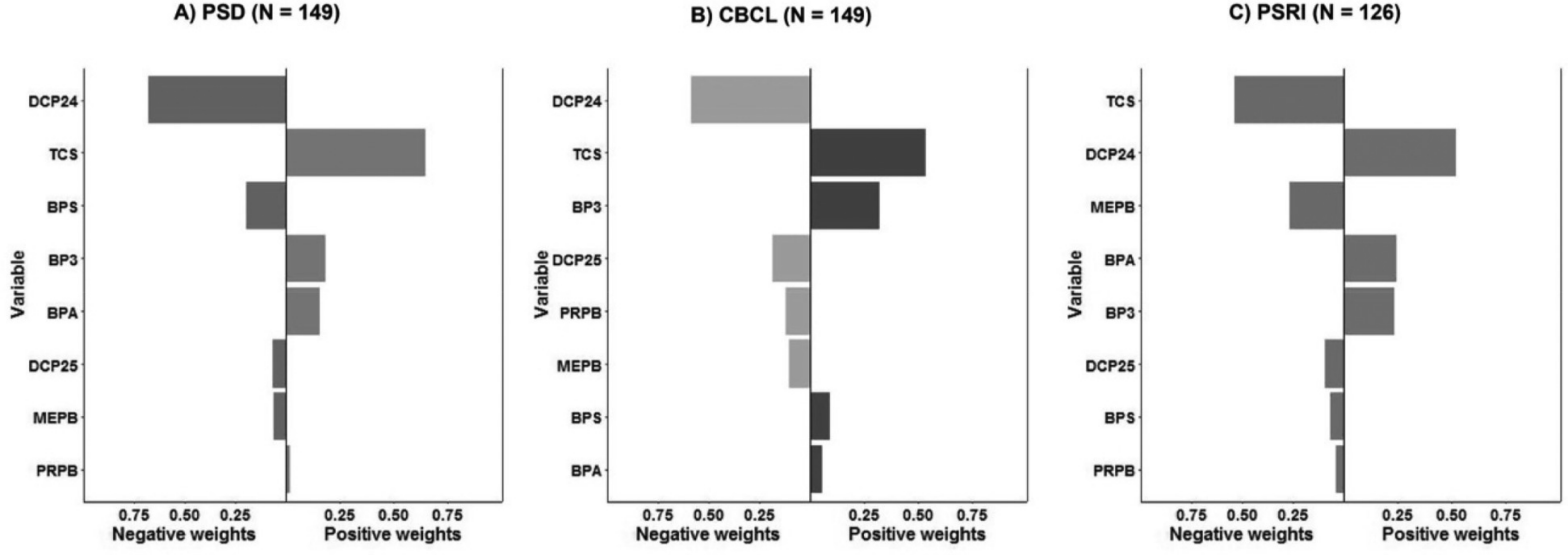
Association between phenol mixture and three child sleep quality outcomes. **(A)** PSD4a (PROMIS Parent Proxy Short Form–Sleep Disturbance 4a); **(B)** CBCL (Child Behavior Checklist Sleep Problems Syndrome Scale); **(C)** PSRI4a (PROMIS Parent Proxy Short Form–Sleep-Related Impairment 4a). Bars represent the weights from quantile g-computation models for each phenol in the mixture; positive and negative weights reflect direction and relative contribution to the sleep outcome.

Table 5 and Figure 1 show the null results of the models assessing the eight phenols as a mixture and child sleep quality. Using quantile g-computation, each simultaneous 1-quartile increase in all phenols together was associated with an increase of 0.47T-score points on the CBCL sleep problems syndrome scale (indicating more sleep problems), but this association was not significant (95% CI: −0.61–1.54). The mixture was slightly negatively associated with PSD4a (*β* = 0.28, 95% CI: −3.12–2.57) and PSRI4a (*β* = −0.07, 95% CI: −3.23–3.10) scores, but the CIs for these associations included the null. We also examined the weights assigned to individual chemicals in the quantile g-computation model. BP3 and methylparaben contributed the largest positive weights for CBCL and PSD4a, consistent with the strongest associations in single-pollutant models. In contrast, BPS and DCP24 contributed minimally or had negative weights, aligning with their weaker individual associations.

**Table 5:** Quantile g-computation estimates and 95% confidence intervals for the change in sleep quality outcomes per one-quartile increase in the phenol mixture.

Overall mixture effect		
Sleep quality	β	95% CI
CBCL	0.47	−0.61–1.54
PSD4a	−0.28	−3.12–2.57
PSRI4a	−0.07	−3.23–3.10

CBCL, Child Behavior Checklist; CI, confidence interval; PSD4a, PROMIS Parent Proxy Short Form-Sleep Disturbance 4a; PSRI4a, PROMIS Parent Proxy Short Form Sleep-related Impairment 4a.

## 4 Discussion

Chemical exposures during pregnancy can impact brain development and hormone levels, adversely affecting health outcomes from infancy into adulthood. However, the link between these chemicals and childhood sleep, an important predictor of childhood and long-term health, is less understood (60, 63, 64). The present analysis helps to address this gap by examining whether exposure to endocrine-disrupting chemicals during pregnancy was associated with parent-reported child sleep measures among a nationally representative, multiethnic cohort sample.

Our sample showed sleep problems and disturbances slightly higher than the US-normed population (mean score of 50) (71), particularly with the largest subsample, CBCL, with a score of 53; this difference could be due in part to the predominance of Hispanic children in our sample. Hispanic children in the U.S. are disproportionately affected by shorter sleep duration and poorer sleep quality, as supported by prior research (72, 73). These disparities may be linked to cultural sleep practices, socioeconomic factors, or environmental stressors that deserve further investigation in future studies.

We found that the direction of the association of prenatal phenol concentration and child sleep quality was positive for most of the single chemicals and for cumulative chemical exposure, indicating that higher phenol exposure was associated with higher child sleep problems, disturbances, and impairments. Findings were somewhat mixed in terms of directionality and dose-response trend, meaning that some associations were inverse and that higher categories of exposure did not always result in stronger effect measures. Interactions with child sex were not consistently significant but suggested that child sex may modify the effect of developmental exposure on child sleep quality.

BP3, a UV-filter commonly used in sunscreens and cosmetics, was the most consistently associated chemical in both single-pollutant and mixture models. BP3 can cross the placental barrier and has been shown to act as an endocrine disruptor, with emerging literature suggesting it may influence thyroid hormone function and neurodevelopmental outcomes in children (74–77). These potential hormonal and neurodevelopmental disruptions may contribute to the altered sleep quality we observed, particularly among boys, though additional sex-stratified research is needed to clarify mechanisms.

We are aware of no prior studies on the association between prenatal environmental phenol exposure and child sleep quality using a chemical mixture method. While several studies have assessed the impact of single chemical exposures on behavioral or neurodevelopmental outcomes, none, to our knowledge, have examined prenatal phenols and sleep-related impairment using mixture models in a national pediatric cohort. Chemical mixture methods approximate real-world exposure more accurately than single-exposure methods, providing a basis for practical implications in relation to a significant public health concern.

The cumulative impacts of developmental phenols on childhood sleep are not well understood. Although we hypothesized an effect of combined exposure to the phenols considered in this analysis on the three sleep quality outcomes, we found no statistically significant associations between prenatal phenol concentrations and child sleep quality. It is possible that of the hundreds of phenols in existence, the limited set analyzed in this study cannot approximate the real-world cumulative effect of exposure and its association with child sleep. Alternatively, it is possible that the differing directions of single-pollutant associations with sleep cancel one another out when considered cumulatively in the mixture model or that residual confounding may be at play.

This study also used parent-reported sleep quality measures, which have the potential for recall bias and bias due to secondhand, rather than direct, reporting. Previous studies have found parent-reported child sleep measures to be reliable; while self-reports can overestimate child sleep duration (83), parents on average overestimate child sleep by only 14 min (84). In pediatric primary care, research shows that parents under-report child sleep problems due to a lack of knowledge about appropriate sleep durations or cultural beliefs about what counts as a medical concern (85). Future studies can seek to mitigate those factors using direct measures of empirical sleep data, such as actigraphy and electroencephalogram, and/or child self-report. Another limitation is the possibility of residual confounding from unmeasured or unavailable variables.

Subsequent studies are needed to further characterize cumulative maternal chemical exposures and their association with sleep quality among children and to explore effect modification by child sex. Better understanding of the developmental (*in utero*) risk factors of poor child sleep is a first step toward developing critical public health and policy interventions to reduce and avoid chemical exposures in pregnant people and mitigate the long-term detrimental health impacts of poor childhood sleep.

## Data Availability

Publicly available datasets were analyzed in this study. These data are available from the NICHD Data and Specimen Hub (DASH) at https://dash.nichd.nih.gov, specifically the ECHO Cohort public-use dataset, which can be accessed directly at https://dash.nichd.nih.gov/study/426432.

## ECHO program collaborators

ECHO Components: Coordinating Center: Duke Clinical Research Institute, Durham, North Carolina: Smith PB, Newby LK; Data Analysis Center: Johns Hopkins University Bloomberg School of Public Health, Baltimore, Maryland: Jacobson LP; Research Triangle Institute, Durham, North Carolina: Catellier DJ; Person-Reported Outcomes Core: Northwestern University, Evanston, Illinois: Gershon R, Cella D.

ECHO Awardees and Cohorts: University of Georgia, Athens, GA: Cordero J; University of Michigan, Ann Arbor, MI: Meeker J; Michigan State University, East Lansing, MI: Kerver JM; Henry Ford Health, Detroit, MI: Barone C; Michigan Department of Health and Human Services, Lansing, MI: Fussman C; Michigan State University, East Lansing, MI: Paneth N; University of Michigan, Ann Arbor, MI: Elliott M; Wayne State University, Detroit, MI: Ruden D; New York University Grossman School of Medicine, New York, NY: Trasande L.
