# Supplementary material for: Environmental phenol mixture during pregnancy and child sleep quality in the ECHO cohort
Source: Front Pediatr. 2025 Aug 8;13:1533015. doi: 10.3389/fped.2025.1533015 (PMC12372471; doi:10.3389/fped.2025.1533015)
Supplement: Supplementary file 1 [file Datasheet1.docx]

Supplementary Material

# Supplementary Figures and Tables

## Supplementary Tables

**Supplementary Table 1. Number of children in the CBCL, PSD4a, and PSRI4a analyses**

| **Cohort name** | **CBCL** | **PSD4a** | **PSRI4a** |
| --- | --- | --- | --- |
|  |  |  |  |
| Archive for Research in Child Health (ARCH) | 88 |  |  |
| Atlanta ECHO Cohort of Emory University | 42 |  |  |
| ECHO in Puerto Rico (PROTECT) | 107 | 126 | 108 |
| Healthy Start | 246 |  |  |
| Illinois Kids Development Study (IKIDS) | 135 | 42 | 34 |
| Pregnancy Environment and Lifestyle Study (PETALS) | 170 | 206 |  |
| The NYU Children’s Health and Environment Study (NYU-CHES) | 335 |  |  |

Abbreviations: CBCL, Child Behavior Checklist; ECHO, Environmental influences on Child Health Outcomes; PSD4a, PROMIS Parent Proxy Short Form-Sleep Disturbance 4a; PSRI4a, PROMIS Parent Proxy Short Form Sleep-related Impairment 4a.

**Supplementary Table 2. Laboratory methods used to measure phenolic compounds across cohorts**

| **Cohort Data** | **N (women)** | **City** | **State** | **Lab name** | **Lab method** | **Sample Description** |
| --- | --- | --- | --- | --- | --- | --- |
| **CBCL** |  |  |  |  |  |  |
| Archive for Research in Child Health (ARCH) | 88 | East Lansing | MI | Wadsworth Center | Liquid chromatography-tandem mass spectrometry (LC-MS/MS) analysis | Pregnant women recruited during their first prenatal visit at 1 of 3 prenatal clinics in Lansing, MI. |
| Atlanta ECHO Cohort of Emory University | 42 | Atlanta | GA | HHEAR, Emory CHEAR Lab | HPLC-MS/MS, solid phase extraction coupled to isotope dilution LC-ESI-MS/MS | African American women recruited from two prenatal clinics in Atlanta, GA, and their offspring. |
| ECHO in Puerto Rico (PROTECT) | 107 | San Juan | PR | CDC, HHEAR | HPLC-MS/MS | Pregnant women recruited from 5 OB/GYN clinics in Puerto Rico and their offspring. |
| Healthy Start | 246 | Aurora | CO | Personal Care Products Laboratory, OATB/DLS/NCEH, CDC | Solid-phase extraction-high performance liquid chromatographic-isotope dilution-tandem mass spectrum | Colorado community sample of mother-child dyads recruited in infancy from obstetrics clinics at a university hospital and by word of mouth, as well as medical university employees. |
| Illinois Kids Development Study (IKIDS) | 135 | Urbana | IL | CDC | NA | Pregnant women recruited from 2 OB/GYN clinics in southern Illinois and their offspring. |
| Pregnancy Environment and Lifestyle Study (PETALS) | 170 | Oakland | CA | CDC Personal Care Products Laboratory, California Dept. of Public Health Environmental Health Laboratory | 6301 (Bisphenol A and Environmental Phenols in Urine), Environmental Phenols in urine by LC/MS/MS | Women identified as pregnant via hospital records from 4 California hospitals recruited before 10 weeks gestation via telephone and their offspring. |
| The NYU Children’s Health and Environment Study (NYU CHES) | 335 | New York | NY | Wadsworth Center New York Department of Health | Shimadzu HPLC system interfaced with API 3200 MS/MS by MRM in the negative ionization mode | Pregnant women recruited from prenatal clinics at NYU Langone Medical Center study sites. |
| **PSD4a** |  |  |  |  |  |  |
| PROTECT | 108 | San Juan | PR | CDC | HPLC-MS/MS | Pregnant women recruited from 5 OB/GYN clinics in Puerto Rico and their offspring. |
| IKIDS | 34 | Urbana | IL | CDC | NA | Pregnant women recruited from 2 OB/GYN clinics in southern Illinois and their offspring. |
| **PSRI4a** |  |  |  |  |  |  |
| PROTECT | 126 | San Juan | PR | CDC | HPLC-MS/MS | Pregnant women recruited from 5 OB/GYN clinics in Puerto Rico and their offspring. |
| IKIDS | 42 | Urbana | IL | CDC | NA | Pregnant women recruited from 2 OB/GYN clinics in southern Illinois and their offspring. |
| PETALS | 206 | Oakland | CA | CDC Personal Care Products Laboratory, California Dept. of Public Health Environmental Health Laboratory | 6301 (Bisphenol A and Environmental Phenols in Urine), Environmental Phenols in urine by LC/MS/MS | Women identified as pregnant via hospital records from 4 California hospitals recruited before 10 weeks gestation via telephone and their offspring. |

Abbreviations: API, atmospheric pressure ionization; CBCL, Child Behavior Checklist; CDC, United States Centers for Disease Control and Prevention; CHEAR, Children's Health Exposure Analysis Resource; ECHO, Environmental influences on Child Health Outcomes; HHEAR, Human Health Exposure Analysis Resource; HPLC-MS/MS, high-performance liquid chromatography-tandem mass spectrometry; LC-ESI-MS/MS, liquid chromatography electrospray ionization tandem mass spectrometry; MRM, multiple reaction monitoring; NYU, New York University; OATB/DLS/NCEH, Organic Analytical Toxicology Branch/ Division of Laboratory Sciences/National Center for Environmental Health; OB/GYN, obstetrics and gynecology PSD4a, PROMIS Parent Proxy Short Form-Sleep Disturbance 4a; PSRI4a, PROMIS Parent Proxy Short Form Sleep-related Impairment 4a.

**Supplementary Table 3. Tertile ranges of phenols for sleep outcome samples overall and by child sex**

|  | **CBCL** | | | **PSD4a** | | | **PSRI4a** | | |
| --- | --- | --- | --- | --- | --- | --- | --- | --- | --- |
| **Phenol (ng/mL for T1, T2)** | **Overall** | **Male** | **Female** | **Overall** | **Male** | **Female** | **Overall** | **Male** | **Female** |
| BP3 | 0.70, 1.44 | 41.76, 246.12 | 36.06, 179.86 | 0.79, 1.61 | 31.49, 227.19 | 35.23, 193.30 | 1.30, 2.04 | 29.30, 104.57 | 29.24, 198.75 |
| BPA | 0.33, 0.75 | 0.70, 1.42 | 0.70, 1.45 | 0.41, 0.91 | 0.83, 1.70 | 0.75, 1.51 | 0.36, 0.75 | 1.33, 1.96 | 1.19, 2.20 |
| BPS | 38.12, 214.56 | 0.34, 0.74 | 0.31, 0.75 | 32.23, 207.12 | 0.42, 0.91 | 0.40, 0.88 | 29.28, 142.39 | 0.38, 0.66 | 0.35, 0.79 |
| DCP24 | 0.46, 1.02 | 0.45, 1.06 | 0.46, 1.00 | 0.63, 1.37 | 0.63, 1.49 | 0.63, 1.32 | 0.63, 1.40 | 0.64, 1.37 | 0.62, 1.40 |
| DCP25 | 1.12, 4.46 | 0.99, 3.98 | 1.30, 4.75 | 3.97, 15.27 | 3.16, 17.88 | 4.71, 12.97 | 4.81, 15.72 | 4.87, 16.00 | 4.81, 14.71 |
| MEPB | 42.71, 171.27 | 41.33, 175.61 | 43.61, 166.11 | 43.56, 183.16 | 45.15, 219.97 | 42.83, 159.21 | 41.26, 182.83 | 44.31, 206.99 | 38.40, 161.47 |
| PRPB | 6.31, 42.98 | 6.96, 44.08 | 5.72, 40.45 | 5.69, 36.69 | 8.55, 47.21 | 5.29, 34.12 | 5.33, 34.61 | 8.53, 38.41 | 5.24, 33.64 |
| TCS | 5.76, 32.60 | 5.45, 28.13 | 6.28, 39.76 | 5.07, 62.26 | 5.12, 39.37 | 4.99, 78.73 | 5.71, 101.86 | 4.99, 40.36 | 6.43, 167.79 |

Abbreviations: BP3, benzophenone-3; BPA, bisphenol A; BPS, bisphenol S; CBCL, Child Behavior Checklist; DCP24, 2,4-dichlorophenol; DCP25, 2,5-dichlorophenol; MEPB, methyl paraben; PSD4a, PROMIS Parent Proxy Short Form-Sleep Disturbance 4a; PRPB, propyl paraben; PSRI4a, PROMIS Parent Proxy Short Form Sleep-related Impairment 4a; t1, Tertile 1; T2, Tertile 2; TCS, triclosan.

## Supplementary Figures

6850 women from 25 cohorts with phenol data in ECHO

1836 participants dropped for specimen collection outside pregnancy

411 dropped due to specimen type blood plasma rather than urine

119 participants dropped due to missing LOD/LOQ values

118 women dropped due to no specific gravity/creatinine data

<5 women with phenol values ≥99% percentile excluded

Children with CBCL pre, PSD4a, or PSRI4a data in ECHO: CBCL Pre N=10,656

PSD4a N=13747, PSRI N=5947

Analytic phenol sample:

4364 mothers from 16 cohorts

Children age <4 or ≥8 years old excluded. CBCL N=1131, PSD4a N=392, PSRI N=151

Phenols with <75% detection rate excluded: 0 participants excluded.

\\\

Mother-child pairs excluded due to having fewer than 10 subjects in one cohort or the child not being the first child of mother.

142 mother-child pairs with both phenol and PSRI4a data for analysis

374 mother-child pairs with both phenol and PSD4a data for analysis

1123 mother-child pairs with both phenol and CBCL presleep data for anaysis

**Supplementary Figure 1. Flow chart of eligibility for the study population by sleep outcome**

CBCL indicates Child Behavior Checklist; ECHO, Environmental influences on Child Health Outcomes; LOD, limit of detection; LOQ, limit of quantification; PSD4a, PROMIS Parent Proxy Short Form-Sleep Disturbance 4a; PSRI4a, PROMIS Parent Proxy Short Form Sleep-related Impairment 4a
